# Molecular insight into the expression of metal transporter genes in *Chryseobacterium* sp. PMSZPI isolated from uranium deposit

**DOI:** 10.1371/journal.pone.0216995

**Published:** 2019-05-23

**Authors:** Macmillan Nongkhlaw, Santa Ram Joshi

**Affiliations:** Department of Biotechnology and Bioinformatics, North Eastern Hill University, Mawlai, Umshing, Shillong, Meghalaya, India; Friedrich Schiller University, GERMANY

## Abstract

Metal tolerant bacterium *Chryseobacterium* sp. PMSZPI previously isolated and characterized from uranium ore deposit was studied for elucidating the role of metal transporter genes belonging to the Cation Diffusion Facilitator (CDF), Root-Nodulation-Division (RND) and P_IB_-type ATPase family in cadmium and uranium tolerance. The bacterium showed tolerance towards cadmium (MIC~6mM) and uranium (MIC~2mM) and was found to harbor metal transporter genes belonging to CDF, RND and P_IB_-type ATPase family of proteins. Expression studies by real-time PCR showed an upregulation of *czcA*(RND), *czcD*(CDF) and *cadA*(P_IB_-type ATPase) genes in presence of cadmium or uranium. Higher expression of *czcA* and *czcD* was found when the bacterium was treated with cadmium and uranium respectively. This study provides significant insight into the molecular mechanism that plays a role in cadmium and uranium tolerance in bacteria.

## Introduction

Metal tolerance is a concerted effort of a number of factors that enables a particular microorganism to survive under stress conditions [1]. One of the known mechanisms is the role of metal transporters that are expressed by the host organism in response to the presence of metals [2]. The role of metal transporters through uptake, efflux or transportation to specialized compartments in a cell is one aspect of maintaining metal homeostasis [3, 4]. In bacteria, metal transporters are mainly found to comprise members of three broad families of proteins; Root Nodulation Diffusion family (RND), Cation diffusion family (CDF) and the P_IB_-type family [2, 5]. Genome-wide studies have revealed that these transporters are widely distributed in bacteria and Archaea [2, 5]. Members from these families have been described to play a role in conferring metal tolerance towards a wide range of divalent metal cations like Cu^2+^, Cd^2+^, Pb^2+^, Co^2+^ and Zn^2+^ [1, 5]. RND proteins are known to efflux metal cations across the membrane while CDF proteins are known to efflux excess divalent cations from the cytoplasm [5–9]. P_IB_-ATPase, on the other hand, has been reported to either export or import metal ion in bacteria [10–12]. Non-essential metals like cadmium (Cd) and uranium (U) are generally found in the environment, albeit at a very low concentration [13, 14]. Cadmium and U concentrations in the soil are approximately around 0.01–1.8 ppm and 1.8 ppm respectively [13, 14]. However, anthropogenic activities accompanying the growth of industries and mining has led to the increase in concentration and distribution of these metals in the environment [13, 14]. Cadmium is found in the environment as divalent cation whereas multivalent elements like U oxidation states depend on the redox conditions and pH of the existing environment.[14–16]. Furthermore, U speciation depends on the presence of competing organic (n-caboxylic acids) and inorganic cations (hydroxide, carbonates, phosphates, sulfates) that readily form complexes with U [16]. Both Cd and U have been reported to affect cellular function in bacteria and other higher life forms through oxidative stress by generation of free radical and reactive oxygen species[17, 18]. Furthermore, Cd have been found to compete with Zn and Mg for their active sites, inhibit heme synthesis and also cause epigenetic changes in DNA expression[19, 20]. Uranium has also been reported to cause DNA damage by causing single stand break and DNA lesions which induce mutation in the gene [21–23]. *Chryseobacterium* sp PMSZPI was isolated from U-deposit located at Domiasiat, India and showed multi-metal tolerant properties and possesses metal transporter belonging to P_IB_-type ATPase (*cadA*) family ([24]. In the present study, the presence of metal transporter genes belonging to RND (*czcA*) and CDF (*czcD*) in *Chryseobacterium* sp. PMSZPI (NCBI Accession No.JF768716) isolated from uranium deposit were investigated to elucidate the role of these transporters in U and Cd stress along with a previously described P_IB_-type ATPase (*cadA*)[24] gene found to occur in this isolate.

## Material and methods

### Screening of metal tolerant genes in PMSZPI

Screening for the presence of metal tolerant genes viz, *czcA* and *czcD* which are members of the RND and CDF family of metal transporting protein respectively in *Chryseobacterium* sp. was carried out using degenerate forward primer and reverse primer shown in S1 Table. These primers were designed using iCODEHOP software [25]. Metal transporter genes were amplified from genomic DNA extracted from the bacterial isolate using Bacterial genomic DNA extraction kit (HiMedia, India). PCR mixtures (25 μL) contained approximately 30ng of template DNA, 2μM forward primer, 2μM reverse primer, Taq DNA Polymerase buffer with 15 mM MgCl_2_, deoxynucleoside triphosphates (250μM each of dATP, dCTP, dGTP and dTTP) and 1.0U of Taq DNA polymerase. DNA amplification was carried out in a GeneAmpPCR system 9700 (Applied Biosystems, USA) with an initial denaturation step of 94°C for 5 min, followed by 30 cycles consisting of denaturation at 94°C for 1 min, annealing at 49°C for 1 min, and extension at 72°C for 1.5 min followed by a final extension step of 72°C for 5 min. The expected amplicons size for *czcD* and *czcA* genes are approximately 450–500 bps and 850–900 bps respectively.

### Sequencing and phylogenetic analysis of *czcA* and *czcD* genes

Amplicons of *czcA* and *czcD* from *Chryseobacterium* sp. were purified using Gel Extraction Kit (HiMedia, India) and sequenced using the Big Dye Terminator cycle sequencing kit v.3.1 (Applied Biosystems, USA) deploying the standard protocol and an automated Genetic Analyzer ABI 3130XL (Applied Biosystems, USA). The Basic Local Alignment Search Tool (BLAST, sub-program BLASTX) was used to determine the phylogenetic neighbors of their respective genes present in GenBank database (National Center for Biotechnology Information, Bethesda, USA)[26]. Phylogenetic analyses were carried out with nucleotide sequences of identified phylogenetic neighbors retrieved from Genbank and aligned using ClustalW of MEGA6 [27]. Phylogenetic tree construction was carried out using neighbor-joining method with 1000 bootstrap replications for nodal support. Phylogenetic analyses based on the maximum likelihood and maximum parsimony of *czcA* and *czcD* amino acid sequences were in agreement with the data generated by the above described neighbor joining method.

### Analysis of minimum inhibitory concentration and viability test

One millilitre of mid-log phase cells (OD_600_ 0.8) of the bacterium grown in LB medium was harvested and washed with sterile distilled water. The washed cells were then resuspended in one millilitre sterile Tris Buffered medium containing 20mM Tris-HCl pH 7.0, 80mM NaCl, 20mM KCl and 5% Glycerol. Varying concentrations of filter sterilized cadmium nitrate or uranyl nitrate solutions were added to each tube and the cell suspensions were incubated at 28°C for three hour in a shaker incubator. Five microlitre was then spotted in LB agar plate and incubated at 28°C for 48 hours. Viability tests of the isolate towards varying concentrations of metals were performed by serial dilutions and plating the inoculums in LB plates. The plates were then incubated at 28°C for 48 hours for observation.

### RNA extraction and cDNA synthesis

Two millilitres of mid-log phase cells (OD_600_ 0.8) grown in LB medium were harvested and washed with sterile distilled water. The washed cells were then resuspended in one millilitre sterile Tris Buffered medium containing 20mM Tris-HCl pH 7.0, 80mM NaCl, 20mM KCl and 5% Glycerol. For metal treatment, 0.5mM of cadmium or uranium was added to the individual cell suspension and incubated for 3 hours at 28°C. Metal treated/untreated cells were then harvested by centrifugation at 10500 X g (HERMLE,Germany) for 2 minutes. Total RNA was extracted using RNeasy Mini Kit (QIAGEN, USA) according to manufacturer’s instructions. On column DNase treatment was performed by adding 100μl of DNase treatment buffer contained 10units of RQ1 DNase (Promega,USA) and incubated for one hour at 37°C. Purified RNA was eluted after DNase treatment in nuclease-free water and quantified by NanoVue (GE Healthcare). For cDNA synthesis, 250ng of RNA was used and reverse transcription was performed using the iScript cDNA Synthesis Kit (BioRad, USA).

### Transcription profiling using real-time PCR

Real-time PCR assays were performed in 10μl reaction volume containing 5μl of 2X GoTaq qPCR Master-mix (Promega, USA), 0.2μl each of forward and reverse primer and 1μl of 10X diluted cDNA. The temperature program for real-time PCR was 95°C for 2 minutes (Hot start activation) followed by 40 cycles of amplification (95°C for 15seconds and 60°C for 60 seconds) for 16S rRNA, *czcA*, *cadA* and *czcD* gene amplification (*cadA* occurrence was previously described in Kumar et al. 2013). The melting curve analysis was performed within the temperature range of 60–95°C. Primers used for real time PCR are listed in Table 1. The Ct values that were obtained from three independent experiments were used for calculating the fold change in expressions using 16S rRNA as an internal control. Statistical analysis was performed using Student’s t-test to determine whether the two dataset were significantly different (p>0.005).

**Table 1:** List of primers for qRT-PCR studies.

Primer	Oligo sequences 5’-3’	Amplicon Size (bp)	Target Gene
ZP1Fc	GGTGGATAGAGATGGGCACG	107	*16S rRNA*
ZP1Rc	AGTACCAGTGTGGGGGATCA		
ZP1F1a	TGATGTTTTCGCAGCATTGG	103	*czcA*
ZP1R1a	TCCCAAAAGTCCCTCACTCC		
PMZF2b	CCCGGTTGCTACTGCAATTC	110	*cadA*
PMZR2b	TTCCATTCACCGTTGCTTTCA		
ZP1F1d	GGAGTCATGATTGCCGGAGTT	120	*czcD*
ZP1R1d	AGCCTCCATCAACAGTTTCCA		

## Results

### Cadmium and uranium tolerance

**Fig 1:**
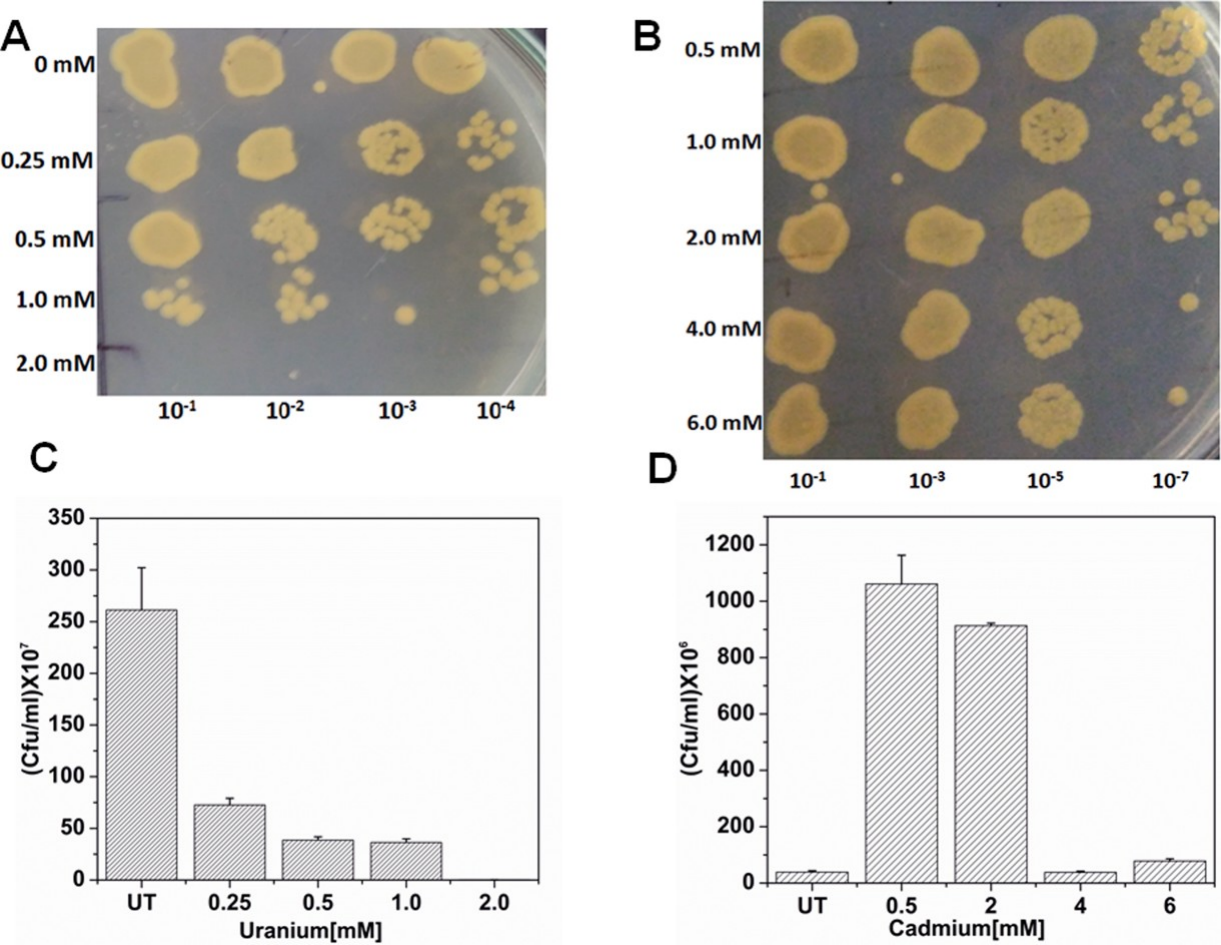
Growth of *Chryseobacterium* sp. after treatment with various concentrations of metal. A) Spot assay to determine the MIC towards Uranium; B) Plating method to determine the effect of U on *Chryseobacterium*sp.; A) Spot assay to determine the MIC towards Cd; B) Plating method to determine the effect of Cd on *Chryseobacterium* sp.

Cadmium and uranium tolerance capabilities of *Chryseobacterium* sp. was monitored by treating the bacterium with increasing concentrations of Cd or U. *Chryseobacterium* sp. showed notable tolerance towards Cd (MIC~6mM) as compared to uranium (MIC~2mM). Cell viability assay showed that Cd has an unusual growth stimulating effect on the bacterium up to a concentration of 2mM beyond which antagonistic effect of metal starts to show on the growth of bacterium (Fig 1D). Uranium, on the other hand, had an adverse effect on the growth of bacterium in the concentration range tested (Fig 1A and 1B).

### Screening for metal tolerant genes

**Fig 2:**
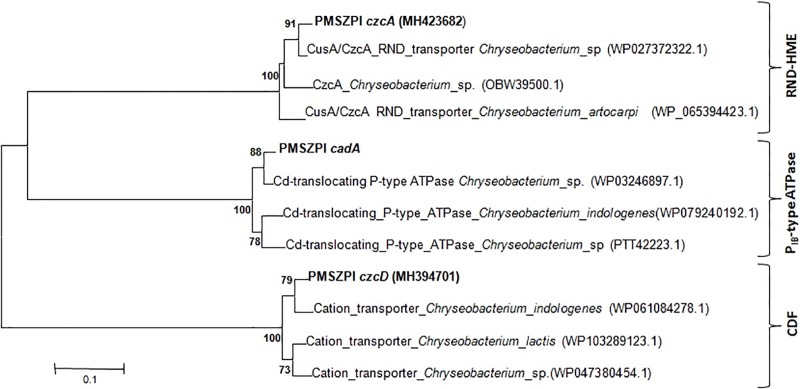
Phylogenetic analysis of CDF, RND-HME and P_IB_-type ATPases. Neighbor-joining method of translated amino acid sequences from *czcD*, *czcA* and *cadA* genes were carried out using MEGA.6.0 software with 1000 bootstrap support.

Metal transporter genes belonging to the member of RND-HME (*czcA*) and CDF (*czcD*) were amplified using primers listed on S1 Table. *Chryseobacterium* sp. PMSZPI was found to harbor all the genes known for the metal tolerant phenotype.BLASTX analyses of the sequenced nucleotide obtained for each amplicon showed that the putative *czcA* and *czcD* amplicons matched their respective genes from other *Chryseobacterium* sp. reported in the NCBI database (Table 2). The similarity percentage of each gene with respect to their close relative reported in the NCBI database is given in Table 2. Phylogenetic analyses of the sequenced amplicons using Neighbor-joining method showed that putative *czcA* and *czcD* genes from *Chryseobacterium* sp.PMSZPI are grouped in their respective gene families (Fig 2).

**Table 2:** BLASTX analysis of metal transporters from *Chryseobacterium* sp. (PMSZPI).

Gene Name (Accession. No)	The closest match of metal tolerant genes from PMSZPI with those in NCBI Database after BLASTX	Similarity Percentage
***czcA***(MH423682)	CusA/CzcA family heavy metal efflux RND transporter [*Chryseobacterium* sp. UNC8MFCol] (WP_027372322.1)	98
***czcD***(MH394701)	Cation diffusion facilitator family transporter [*Chryseobacterium* sp. ISE14] (WP109713444.1)	97
***cadA***(JN034431)	Cadmium-translocating P-type ATPase [*Chryseobacterium* sp. ISE14] (WP_103246897.1)	98

### Expression of *czcA*, *czcD* and *cadA*in the presence of Cd and U

**Fig 3:**
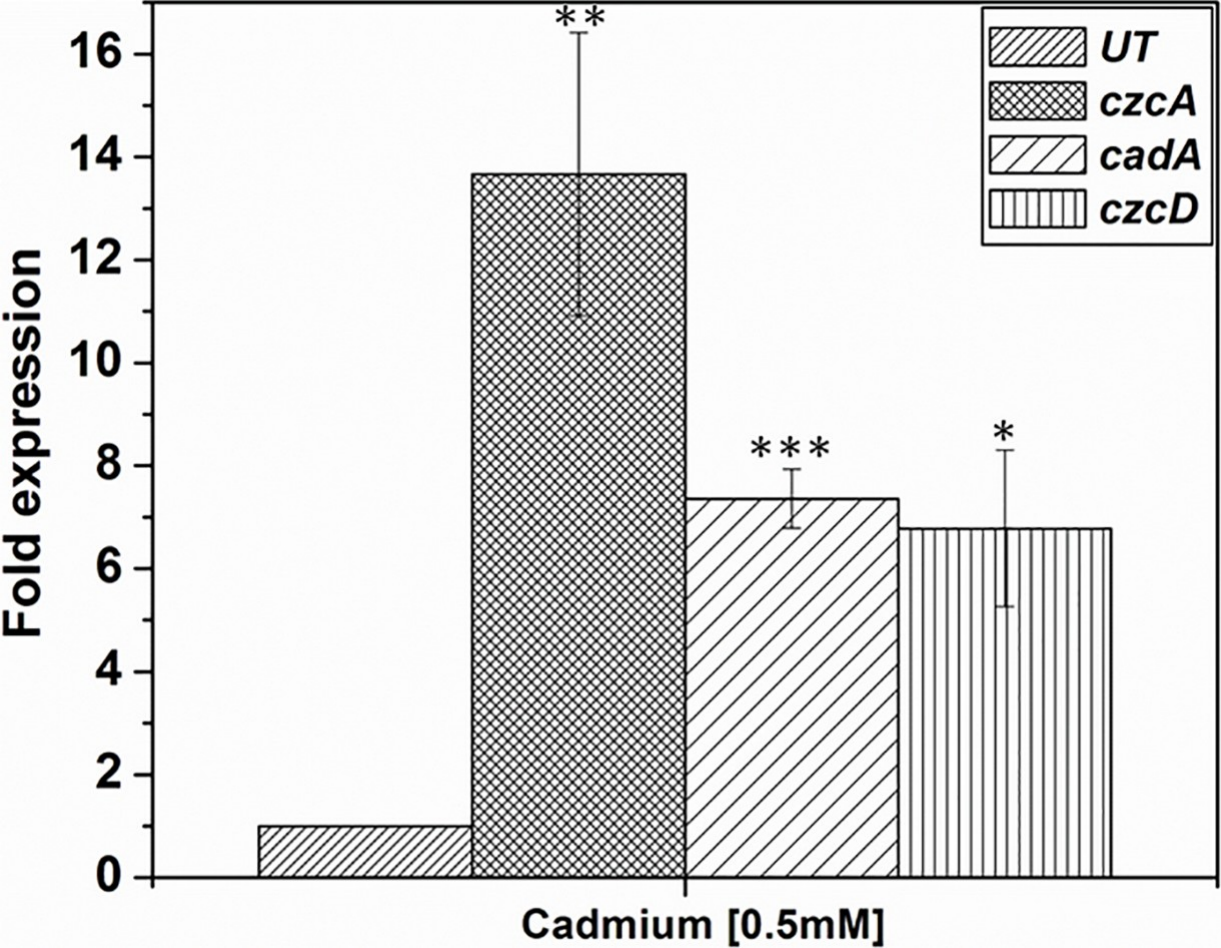
Expression of metal transporter genes in the presence of cadmium. Expression of *czcA*, *czcD* and *cadA* genes in response to treatment with 0.5mM Cd. Expression of these genes was normalized to the expression of the 16SrRNA gene. Statistical significance was performed using Student’s test (p ≤0.05*; p ≤0.01≥0.001 **; p ≤0.001***.).

**Fig 4:**
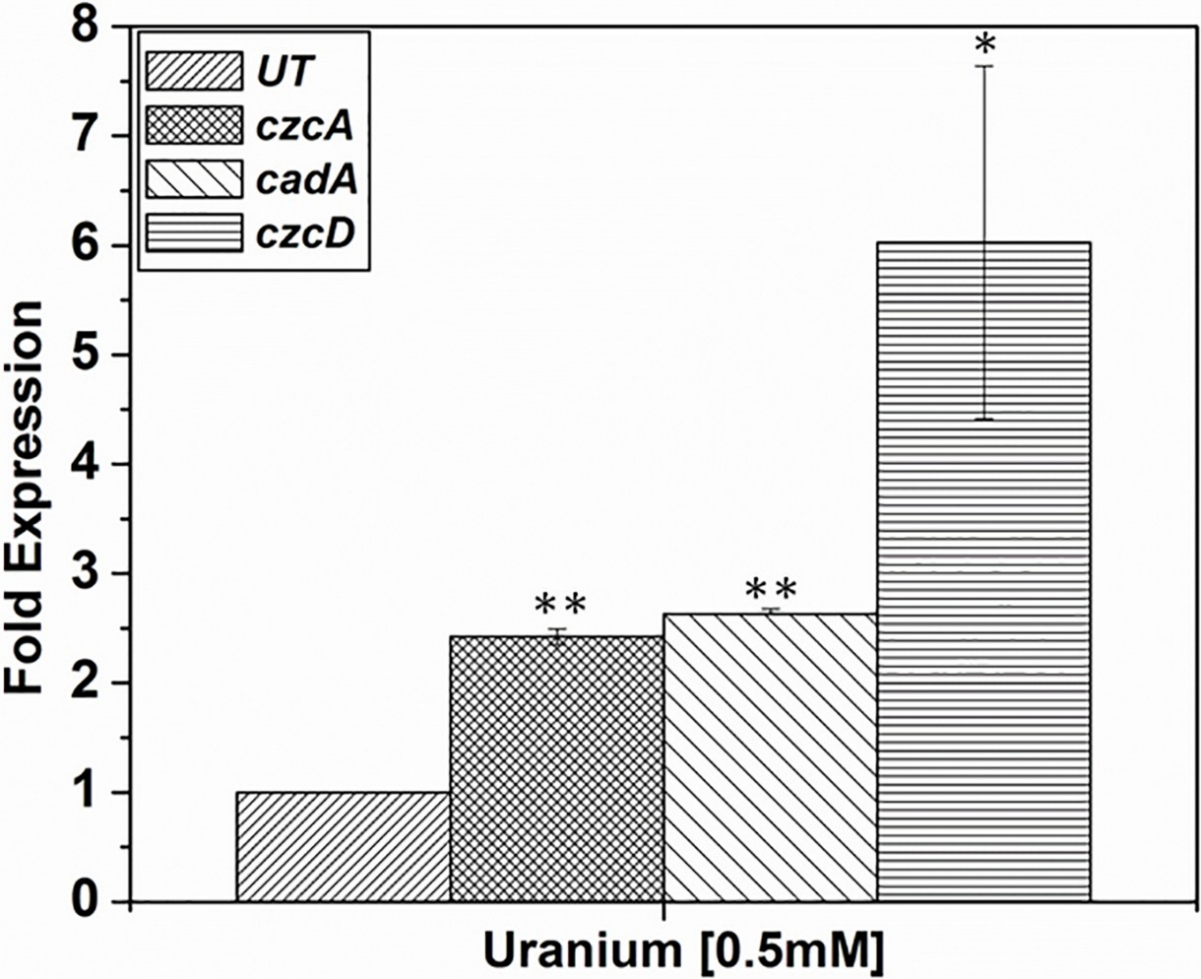
Expression of metal transporter genes in the presence of uranium. Expression of *czcA*, *czcD* and *cadA* genes in response to treatment with 0.5mM U. Expression of these genes was normalized to the expression of the 16SrRNA gene. Statistical significance was performed using Student’s test (p ≤0.05 *; p ≤0.01≥0.001 **; p ≤0.001 ***.).

Previously described *cadA* gene belonging to the family of P_IB_-type ATPase from *Chryseobacterium* sp. PMSZPI [28] along with the newly identified *czcA* and *czcD* genes belonging to the RND family and CDF family of metal transporters were studied for their role in U or Cd stress. Expression of metal tolerant genes was studied by monitoring the level of mRNA transcripts of *czcA*, *czcD* and *cadA* genes in the presence of Cd or U. The level of expression was normalized to the expression of 16S rRNA both in treated/untreated cell. In this study, all the three genes under study were found to be upregulated in the presence of Cd or U. In this study, *czcA cadA* and *czcD* gene expressions were found to be 13, 7 and 6-fold upregulated respectively in the presence of 0.5mM Cd (Fig 3). Similarly, *czcA* and *cadA* genes were found to be 2-fold upregulated and *czcD* was 6-fold upregulated in the presence of 0.5mM U (Fig 4).

### Nucleotide sequence accession number

The GenBank accession numbers obtained for *czcA* and *czcD* genes reported in this study are MH423682 and MH394701 respectively.

## Discussion

Metal tolerant bacteria have been isolated from naturally occurring or anthropogenic metal-rich environment for their use in bioremediation, agriculture or understanding the mechanism for their survival in such extreme habitat [24, 28–31]. These isolates tend to possess additional features that allow them to exist in such extreme habitat [29]. *Chryseobacterium* sp. PMSZPI was previously isolated from uranium deposit and identified using biochemical and molecular methods [24]. PMSZPI showed significant tolerance towards U and Cd as evident from MIC and viability studies (Fig 1). This isolate was also found to tolerate high concentration of copper and zinc as reported in earlier studies [24]. Interestingly, the growth of PMSZPI seems to increase with the treatment of low concentration of Cd, even though it is known that Cd have no essential biological role (Fig 1D). Metal tolerance is a concerted effort of various molecular actors that maintain the metal homeostasis of both essential and non-essential metals that enter the cytoplasm of the microorganism [7, 32, 33]. Cadmium toxicity in living organisms is a result of its incursion into various molecular processes. One of the molecular mechanism to decrease Cd-toxicity is the involvement of metal efflux proteins[2, 34]). PMSZPI was found to harbor all the three member of metal efflux proteins that are known to play a major role in maintaining the cellular metal homeostasis in bacteria [2]. The three metal transporters were phylogenetically distinct from each other as evident from BLASTX and Phylogenetic analyses (Table 2 and Fig 2). Metals tolerant genes belonging to the family of CDF (*czcD*), RND-HME(*czcA*) and P_IB_-type ATPase (*cadA*) have been studied for their role in Cd^2+^ Cu^+/2+^ Zn^2+^ Pb^2+^ tolerance in bacteria[2]. Similarly, *czcD*, *czcA* and *cadA* genes from PMSZPI were found to be upregulated in response to Cd treatment indicating that these transporters have a role in Cd tolerance. Previous studies showed that there is the interplay between the czcCBA efflux system, P_IB_-type ATPases and CDF protein in *Ralstonia metallidurans* where the former was found to play a major role in metal detoxification [7, 8]. Similarly, in this study, we found that *czcA* expression was 2-fold higher as compared to *cadA* and *czcD* expression in the presence of Cd indicating that czcCBA efflux system might play a major role in Cd tolerance in this isolate. The interaction of U with various life forms is a widely studied area due to the recent increase in U exploration for its use in energy generation and weaponry. Understanding uranium toxicity is one the aspect that most researchers are trying to elucidate by studying its molecular interactions with biomolecules and their metabolic processes [16]. The mechanisms of Utolerance in bacteria are carried out through various biological processes like bioreduction, biomineralization, biosorption and bioaccumulation where they have been reported to play a major role in decreasing the toxicity of U [35]. Recent studies have been carried out to understand the molecular response towards uranium’s interaction with bacterial cells. These studies included proteomic analysis [36, 37], transposon-mediated mutagenesis [38] and whole genome transcriptional and functional analysis of genes in a microorganism that showed U tolerance capabilities[39–41]. These studies revealed the expression of genes involved in bioprecipitation, metal efflux, and oxidative stress besides other genes involved in cell metabolism. In this study, increase in the expression of metal efflux proteins belonging to the member of RND-HME, CDF and P_IB_-type ATPase was seen during U treatment indicating that these transporters play a part in U tolerance in *Chryseobacterium* sp. Furthermore, there is a 6-fold upregulation of *czcD* gene as compared to *cadA* and *czcA* during U treatment indicating that CDF protein may have a primary role in U tolerance. As expected, CDF, RND-HME and P_IB_-type ATPases which are known to play a major role in Cd tolerance in other microorganisms showed higher expressions when treated with Cd as compared to U. The findings of the present study revealed that these efflux proteins in *Chryseobacterium* sp. are also involved in U tolerances. However, toxicity of U depends on its speciation which is determined by the physicochemical components of that ecosystem. So, one of the major challenges in studying U interaction with biomolecules *in situ* is its speciation and complex formation in natural conditions. Nonetheless, this study showed that metal efflux proteins were found to respond toward U stress indicating that they may play a role either directly or indirectly in U tolerance.

## Compliance with ethical standards

The article does not contain any studies with human participants or animals performed by any of the authors.

## Supporting information

S1 Table List of primers used for screening metal tolerant genes in *Chrysobacterium* sp. PMSZPI. (PDF)
