# Supplementary material for: Molecular insight into the expression of metal transporter genes in Chryseobacterium sp. PMSZPI isolated from uranium deposit
Source: PLoS One. 2019 May 23;14(5):e0216995. doi: 10.1371/journal.pone.0216995 (PMC6532875; doi:10.1371/journal.pone.0216995)
Supplement: S1 Table — (PDF) [file pone.0216995.s001.pdf]

S1 Table. List of primers used for screening metal tolerant genes in *Chrysobacterium* sp. PMSZPI

Primer	Oligo sequences 5'-3'	Target Genes (Organisms)	Annealing Tm (ºC)
czcAF1	CGACCTGCGCACCNTNCARGAYTG	*czcA* gene (*Chryseobacterium* sp., *Serratia* sp., *Pseudomonas* sp.)	46
czcAR1	CGGGTGGAACATCTTGCCYTCNWSNCC		
czcDF2	CGATGCCGGCCATATGYTNWSNGA	*czcD* gene (*Arthrobacter* sp.)	49
czcDR2	CGGATGTAATTGTCCAAATATGNARRTCRTG		
81JC	GGATGTCCTTGTGCTYTART	*cadA* gene (*Bacillus, Acinobacter, Pseudomonas*)(Martinez et al.2006)	49
84JC	GGAGCATCGTTAATDCCRTCDCC		

Table 2: List of primers for qRT-PCR studies

Primer	Oligo sequences 5’-3’	Amplicon Size (bp)	Target Gene
ZP1Fc	GGTGGATAGAGATGGGCACG	107	*16S rRNA*
ZP1Rc	AGTACCAGTGTGGGGGATCA		
ZP1F1a	TGATGTTTTCGCAGCATTGG	103	*czcA*
ZP1R1a	TCCCAAAAGTCCCTCACTCC		
PMZF2b	CCCGGTTGCTACTGCAATTC	110	*cadA*
PMZR2b	TTCCATTCACCGTTGCTTTCA		
ZP1F1d	GGAGTCATGATTGCCGGAGTT	120	*czcD*
ZP1R1d	AGCCTCCATCAACAGTTTCCA		

Table 3: BLASTX analysis of metal transporters from *Chryseobacterium* sp. (PMSZPI)

Gene Name (Accession. No)	The closest match of metal tolerant genes from PMSZPI with those in NCBI Database after BLASTX	Similarity Percentage
czcA(MH423682)	CusA/CzcA family heavy metal efflux RND transporter [*Chryseobacterium* sp. UNC8MFCol] (WP_027372322.1)	98
czcD(MH394701)	Cation diffusion facilitator family transporter [*Chryseobacterium* sp. ISE14] (WP109713444.1)	97
cadA(JN034431)	Cadmium-translocating P-type ATPase [*Chryseobacterium* sp. ISE14] (WP_103246897.1)	98
